# Comparative Efficacy and Safety of Anti-PD-1/PD-L1 for the Treatment of Non-Small Cell Lung Cancer: A Network Meta-Analysis of 13 Randomized Controlled Studies

**DOI:** 10.3389/fonc.2022.827050

**Published:** 2022-05-10

**Authors:** Maofen Jiang, Chunjiao Liu, Dongxiao Ding, Hui Tian, Chaoqun Yu

**Affiliations:** ^1^Department of Pathology, Beilun District People’s Hospital, Ningbo, China; ^2^Department of General Thoracic Surgery, Beilun District People’s Hospital, Ningbo, China; ^3^Department of General Thoracic Surgery, Ningbo Medical Center Lihuili Hospital, Ningbo, China

**Keywords:** non-small cell lung cancer, chemotherapy, programmed cell death 1, programmed cell death ligand 1, network meta-analysis

## Abstract

**Objective:**

The present network meta-analysis (NMA) was conducted to summarize the direct and indirect evidence of common programmed cell death 1 (PD-1)/programmed cell death ligand 1 (PD-L1) inhibitors including avelumab, atezolizumab, cemiplimab, nivolumab, and pembrolizumab for the treatment of non-small cell lung cancer (NSCLC) patients and further to determine the optimal therapeutic regimen.

**Methods:**

We performed a systematic literature search to identify all potentially eligible studies in PubMed, Embase, and the Cochrane Library until August 7, 2021. The primary outcome was overall survival (OS), and the second outcome was treatment-related adverse events (TRAEs). We used random-effects model to conduct direct and network meta-analyses, which were performed by using RevMan 5.3 and R version 3.6.1, respectively.

**Results:**

Direct meta-analysis suggested that atezolizumab, cemiplimab, nivolumab, or pembrolizumab significantly improved OS compared with chemotherapy (CT), and NMA further established that atezolizumab [hazard ratio (HR), 0.77; 95% CrI, 0.62–0.96], nivolumab (HR, 0.75; 95% CrI, 0.62–0.93), or pembrolizumab (HR, 0.71; 95% Credible interval (Crl), 0.57–0.89) significantly and cemiplimab (HR, 0.68; 95% CrI, 0.46–1.02) numerically improved OS compared with CT. Meanwhile, NMA also indicated that cemiplimab was numerically superior to other PD-1/PD-L1 agents. Moreover, avelumab, atezolizumab, cemiplimab, nivolumab, and pembrolizumab were found to have fewer TRAEs compared with CT in direct meta-analysis, which were supported by the results from the NMA. Meanwhile, surface under the cumulative ranking curve (SUCRA) and ranking probability suggested that cemiplimab provided the most favorable balance between efficacy and safety, with the first ranking for the OS.

**Conclusions:**

Based on available evidence, cemiplimab may have the most favorable risk–benefit ratio for NSCLC patients compared with other common therapeutic management. However, future research with a large-scale, high-quality, and mature follow-up is needed to further determine which agents should be preferentially selected for NSCLC patients due to the limitations of our NMA and variations of eligible studies in treatment line and PD-L1 status.

## Introduction

According to the Global Cancer Statistics 2020, lung cancer is estimated to have 2.20 million new cases and 1.80 deaths, ranking at the second and first place among all types of cancers, respectively (1). As the most common type of lung cancer, non-small cell lung cancer (NSCLC) accounts for approximately 85% of all lung cancer cases (2). Chemotherapy (CT) has been extensively used as the first-line treatment for patients with NSCLC (3). A meta-analysis comparing CT with supportive care exclusively in advanced NSCLC demonstrated a benefit to CT in the reduction of risk of death and an improvement in 1-year survival (4). Even so, this conventional treatment is still limited due to poor prognosis and several side effects, such as neurologic and renal toxicities and treatment-related nausea and vomiting (5–7). Considering these conditions, novel and effective therapeutic methods are urgently needed.

Over the past decade, the advent of immunotherapy has revolutionized the treatment paradigm of advanced NSCLC. Different from other treatment strategies, immunotherapy regulates the immune system and opens up possibilities for long-term survival outcomes with superior tolerability (8). As a part of immunotherapy, inhibitors of programmed cell death 1 (PD-1) and programmed cell death ligand 1 (PD-L1) such as avelumab, atezolizumab, cemiplimab, durvalumab, nivolumab, and pembrolizumab have been proven to be promising therapeutic options for patients with NSCLC (9–14). These immune checkpoint inhibitors (ICIs) block PD-1/PD-L1 interaction with anti-PD-1/PD-L1 monoclonal antibodies and thus unleash immune response against cancer cells and accelerate the death of tumor cells (8, 15–17). Among them, atezolizumab, durvalumab, nivolumab, and pembrolizumab were approved as standard treatment options for pretreated NSCLC patients, and cemiplimab was approved as the first-line treatment of NSCLC with high PD-L1 expression (≥50%) in February 2021. All of these agents achieved superior overall survival (OS) and less toxicities compared to conventional CT (14, 18–28). However, avelumab may have efficacy similar to or greater than that of those ICIs mentioned above (12). In advance NSCLC population with higher PD-L1 of at least 80%, a *post hoc* analysis revealed that 2-year OS rates were doubled with avelumab (40.2%) compared with docetaxel (20.3%) (13).

It is crucial to recognize any differences in both efficacy and toxicity profiles that may assist clinicians to select the best drug for each patient. However, the limitations of traditional meta-analysis and the lack of direct comparisons comparing all of these ICIs simultaneously leave uncertainty regarding the most effective regimen for NSCLC patients. To investigate this important question, we used a network meta-analysis (NMA) approach to quantify the relative efficacy of regimens that have not been compared within direct comparisons and rank multiple regimens (29). While fully respecting randomization of the included trials, this method conducts a unified coherent analysis of all relevant randomized controlled trials (RCTs). The current NMA summarized the direct and indirect evidence for different PD-1/PD-L1 inhibitors and aimed to determine the optimal therapeutic regimen for NSCLC. The primary endpoint is OS, and the second one is treatment-related adverse events (TRAEs).

## Methods

### Study Design

We performed this NMA in accordance with the Preferred Reporting Items for Systematic Reviews and Meta-Analyses for NMA (PRISMA-NMA) guidelines (30). The completed PRISMA-NMA checklist was available at **Table S1**. Moreover, the formal protocol of this NMA was not registered at a public platform. No patient’s informed consent and ethical approval was required because all analyses were based on data from published studies.

### Information Sources

We systematically searched PubMed, Embase, and the Cochrane Library for RCTs published before August 7, 2021, without language or date restrictions. The search terms used were “carcinoma, non-small-cell lung,” “immune checkpoint inhibitors,” “programmed cell death 1 receptor,” “B7-H1 antigen,” “CTLA-4 antigen,” “drug-related side effects and adverse reactions,” “avelumab,” “atezolizumab,” “cemiplimab,” “durvalumab,” “nivolumab,” “pembrolizumab,” and the name of other PD-1/PD-L1 inhibitors. See **Table S2** for full search strategies.

### Selection Criteria

We included trials that had compared the efficacy of PD-1/PD-L1 inhibitors as monotherapy in patients with NSCLC. The following inclusion criteria were predefined: (a) adult patients had histologically confirmed previously NSCLC (aged ≥18 years); (b) there are three main treatment arms in the trial: anti-PD-1, anti-PD-L1, and CT; each arm should only contain one medication; (c) the outcome is OS and TRAEs, and only trial reports providing data on OS were eligible, regardless of follow-up length; (d) only phase II/III, RCTs with full-text were included; and (e) in cases of duplicate publications, only the most recent and updated publication was included.

We excluded studies if they did not provide enough data to obtain hazard ratios (HRs) for survival. Trials for which full-text reports were not available were also excluded. Furthermore, crossover trials, non-randomized trials, ongoing studies, and observational studies were also excluded in this analysis in order to minimize the risk of bias.

### Study Selection

Two reviewers independently evaluated and screened titles and abstracts grounded on predefined inclusion and exclusion criteria. And then, they retrieved full texts of all potentially relevant studies for further checking eligibility. Additionally, we manually searched bibliographies of the retrieved literature to ensure that no potential trials were missing. If necessary, any discrepancies were resolved by seeking a decision from a third researcher.

### Definition of Outcome

We prespecified OS as the primary outcome. Secondary outcome measure was TRAEs. To match the definitions used in the original studies, OS was defined as the time from randomization to death with any cause. TRAEs were defined and graded according to the National Cancer Institute Common Terminology Criteria for Adverse Events.

### Data Extraction

After reading the original literature, two different investigators extracted the following information: (a) details of the studies: first author, publication year, national clinical trial number, study design, trial phase, recruiting areas, therapy line, the type of ICI drugs, the randomized number of patients, treatment regimens, follow-up time, funding source; (b) population characteristics including tumor grade, tumor histology, and median age; (c) reported outcomes: OS and TRAEs. The variables of interest were HRs with 95% confidence intervals (CIs) for OS and odds ratio (OR) for TRAEs. Furthermore, we also extracted information about the quality of the included studies. Any divergence was solved by discussion with another author.

### Risk of Bias Assessment

The methodological quality of the selected studies was assessed by using the Cochrane risk of bias assessment tool (31). The following items were summarized including random sequence generation; selective outcome reporting; blinding of participants, personnel, and outcome assessors; incomplete outcome data; allocation concealment; and other biases. Each item was labeled as low, unclear, or high risk of bias according to the evaluation criteria (31). Two independent reviewers completed the above tasks, and discordance was solved by consensus.

### Geometry of the Network

Network plots were produced to visualize the body of available evidence. In network geometry, nodes represent the interventions, and their sizes are proportional to the total sample size; lines between the nodes represent direct comparisons, and the thickness of the lines correlate to the number of RCTs evaluating these ICIs.

### Statistical Analysis

Pooled HRs with 95% CIs were calculated for OS, and pooled ORs with 95% CI were calculated for the rate of TRAEs. For OS, we retrieved HRs and the corresponding 95% CI from original studies. If these variables were not available directly, we estimated the HRs using the reported median OS times and P values from log-rank tests or applied the Engauge Digitizer v4.1 software to obtain the time-to-event data from the survival curves (32, 33). Specifically, the natural logarithm of HR (lnHR) and standard error (SE) were computed for subsequent analysis (32, 34). Then, we adopted the pertinent graph-theoretical method to conduct NMA using the transformed HRs and the corresponding SEs from different studies (35). For TRAEs, we calculated ORs by reckoning the number of patients suffering TRAEs by the number of TRAEs (i.e., assuming 1 adverse event per patient). If author only mentioned percentages, we calculated the number of TRAEs by multiplying percentages by the number of patients. We simultaneously used the Cochrane’s Q and the inconsistency statistic (I^2^) to describe heterogeneity across studies (36). Considering that variations across in the real settings are unlikely to be eliminated, all statistical analyses were conducted at the basis of random-effects model regardless of the level of statistical heterogeneity.

For each endpoint, a Bayesian NMA in random-effects model was performed to combine both direct and indirect evidence (37, 38). Treatment effects were estimated by calculating HRs or ORs with corresponding 95% CIs (39, 40). Each ICI was ranked using the surface under the cumulative ranking curve (SUCRA), and a treatment hierarchy was generated (29). A treatment ranked 100% is certain to be the best, and a treatment ranked 0% is certain to be the worst (29, 41). Similarity assumption was examined by assessing studies that compared two interventions and evaluating direct and indirect comparisons (42). In our study, all eligible studies were designed to have two arms that compared experimental treatments with CT, so only shape evidence-structure was available. That means that the distribution of potential confounders and effect modifiers is similar across different pairs of comparisons within the whole network (43), so we did not check the local inconsistency of the NMA.

All statistical analyses were performed using RevMan 5.3 (used for pairwise meta-analysis) and R version 3.6.1 (used for conducting NMA with gemtc package, assessing global heterogeneity and calculating SUCRA).

## Results

### Study Selection and Characteristics

**Figure 1:**
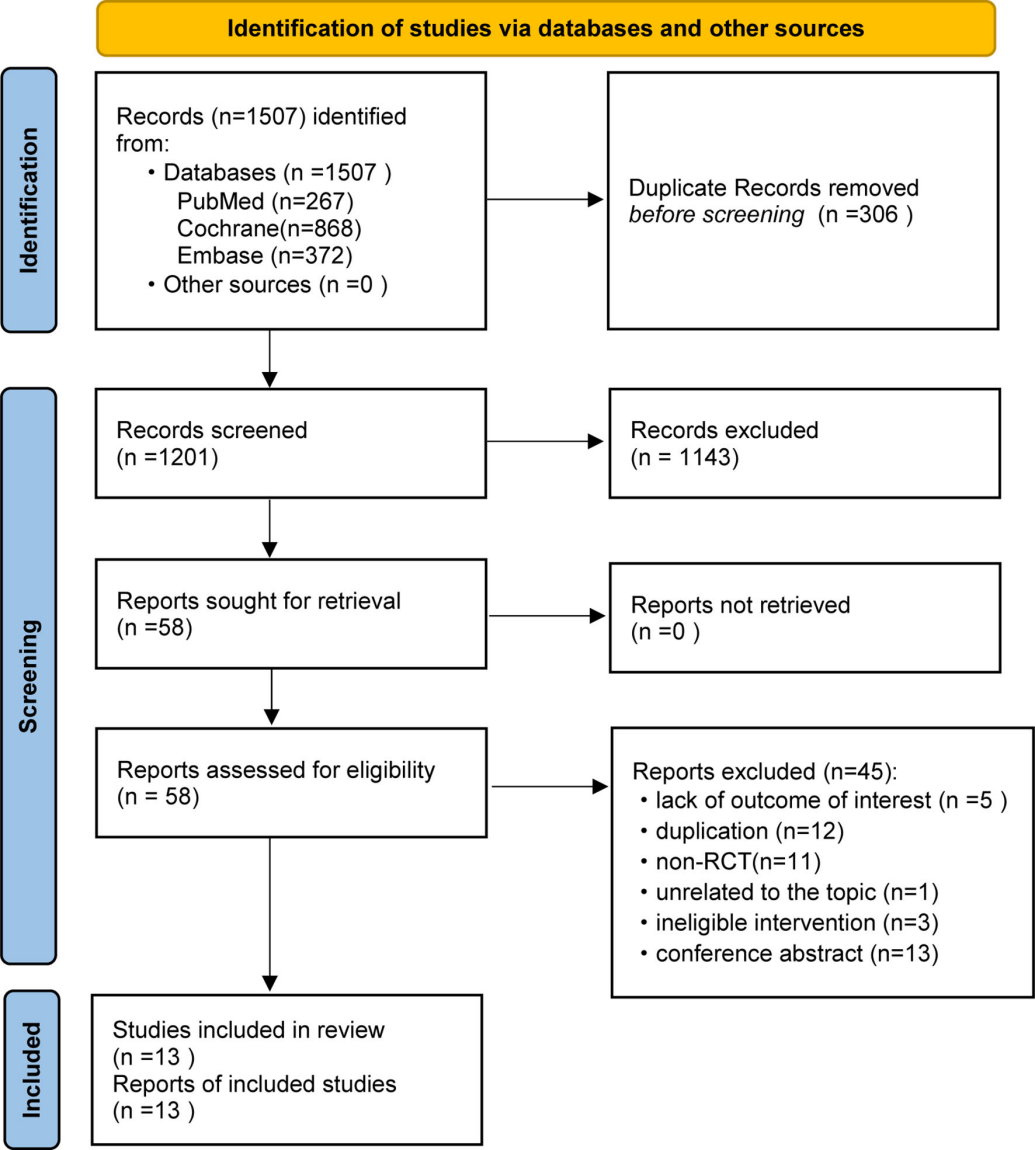
Preferred Reporting Items for Systematic Reviews and Meta-Analyses (PRISMA) flow diagram of retrieval and selection of studies.

We identified 1,507 potentially relevant articles for review of the title and abstract. Eventually, a total of 13 RCTs fulfilled the selection criteria and were included for NMA. All included studies were published as full articles. The details of our literature search are shown in **Figure 1**.

**Figure 2:**
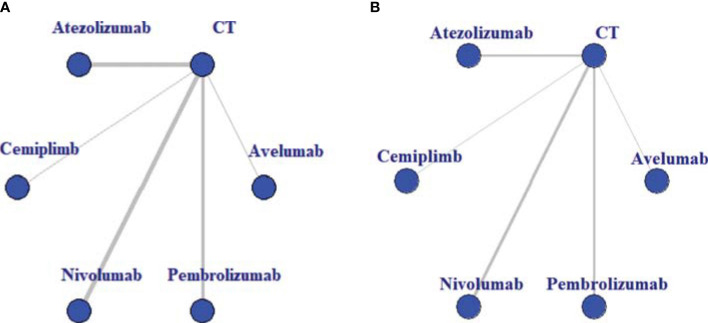
Evidence structure of overall survival **(A)** and treatment-related adverse events **(B)**. All immune checkpoint inhibitors (ICIs) are represented as blue solid circles, and existing head-to-head (direct) comparisons are drawn as black solid lines. The size of every node is proportional to the number of randomly assigned participants (sample size), and the width of the lines is proportional to the number of randomized controlled trials (RCTs) for each pairwise comparison. CT, chemotherapy.

The baseline characteristics of included articles are summarized in **Table 1**. Publication year was between 2015 and 2020, and the number of participants of individual studies ranged from 73 to 1,274. Among them, one trial exclusively enrolled patients with non-squamous NSCLC (28), and one with squamous NSCLC (27). All trials included a CT control arm. Experimental arms consisted of avelumab (n = 1) (13), atezolizumab (n = 4) (21, 22, 24, 26), cemiplimab (n = 1) (14), nivolumab (n = 4) (19, 25, 27, 28), or pembrolizumab (n = 3) (18, 20, 23). In total, our analysis included 7,795 patients, with 369 in avelumab, 903 in atezolizumab, 356 in cemiplimab, 1,036 in nivolumab, 1,480 in pembrolizumab, and 3,624 in CT group. Avelumab and cemiplimab were less frequently investigated by researchers, including fewer sample sizes. This issue revealed a higher potential deviation in head-to-head meta-analysis. To illustrate the head-to-head comparisons involved in the NMA, network plots for OS and TRAEs were delineated in **Figures 2A, B**, respectively.

**Table 1:** Characteristics of the 13 trials included in the network meta-analysis.

Reference	Registry number	Study design	Therapy regimen	Tumor stage	ICI drug	Histology	Age, years(median)	Number of patients randomized(Exp/Con)	Treatments	Follow-up (months)	Funding/Support
Park 2021 (13)	NCT02395172	Multicenter, phase 3	Second-line	IIIB or IV	PD-L1	Non-squamous and squamous	64/63	396/396	10 mg/kg avelumab every 2 weeks or 75 mg/m^2^ docetaxel, every 3 weeks	26+	EMD Serono Research & Development Institute
Fehrenbacher 2016 (26)	NCT01903993	Multicenter, phase 2	Second- and third-line	n.r.	PD-L1	Non-squamous and squamous	62/62	144/143	1,200 mg Atezolizumab or 75 mg/m^2^ docetaxel every 3 weeks	14.8+	F. Hoffmann–La Roche/Genentech
Herbst 2020 (21)	NCT02409342	Multicenter, phase 3	First-line	IV	PD-L1	Non-squamous and squamous	64/65	285/287	1,200 mg Atezolizumab or platinum-based chemotherapy every 3 weeks	8+	F. Hoffmann-La Roche/Genentech
Pujol 2019 (22)	NCT03059667	Multicenter, phase 2	Second-line	n.r.	PD-L1	n.r.	65.9/63.5	49/24	1,200 mg Atezolizumab or 1,200 mg conventional chemotherapy every 3 weeks	13.7	Intergroupe Francophone de Cancérologie Thoracique and Roche SA France
Rittmeyer 2017 (24)	NCT02008227	Multicenter, phase 3	Second-line	IIIB or IV	PD-L1	Non-squamous and squamous	63/64	425/425	Atezolizumab or 75 mg/m^2^ docetaxel every 3 weeks	21	F. Hoffmann-La Roche/Genentech
Sezer 2021 (14)	NCT03088540	Multicenter, phase 3	First-line	IIIB, IIIC or IV	PD-L1	Non-squamous and squamous	63/64	356/354	350 mg cemiplimab or platinum-doublet chemotherapy every 3 weeks	10·8+	Regeneron Pharmaceuticals and Sanofi
Borghaei 2015 (28)	NCT01673867	Multicenter, phase 3	Second-line	IIIB or IV	PD-1	Non-squamous	61/64	292/290	3 mg/kg nivolumab every 2 weeks or 75 mg/m^2^ docetaxel every 3 weeks	13.2+	Bristol-Myers Squibb
Brahmer 2015 (27)	NCT01642004	Multicenter, phase 3	Second-line	IIIB or IV	PD-1	Squamous	62/64	135/137	3 mg/kg nivolumab every 2 weeks or 75 mg/m^2^ docetaxel every 3 weeks	11+	Bristol-Myers Squibb
Carbone 2017 (25)	NCT02041533	Multicenter, phase 3,	First-line	IV	PD-1	Non-squamous and squamous	63/65	271/270	3 mg/kg nivolumab every 2 weeks or platinum-based doublet chemotherapy every 3 weeks	13.5	Bristol-Myers Squibb
Lu 2021 (19)	NCT02613507	Multicenter, phase 3	Second-line	IIIB or IV	PD-1	Non-squamous and squamous	60/60	338/166	3 mg/kg nivolumab every 2 weeks or 75 mg/m^2^ docetaxel every 3 weeks	25.9+	Bristol-Myers Squibb
Herbst 2021 (20)	NCT01905657	Multicenter, phase 2/3	Second-line	IIIB or IV	PD-1	Non-squamous and squamous	63/62	690/343	2 mg/kg or 10 mg/kg pembrolizumab or 75 mg/m^2^ docetaxel every 3 weeks	67.4	Merck & Co.
Mok 2019 (23)	NCT02220894	Multicenter, phase 3	First-line	n.r.	PD-1	Non-squamous and squamous	63/63	637/637	200 mg pembrolizumab or platinum-based chemotherapy every 3 weeks	12.8	Merck Sharp & Dohme
Reck 2021 (18)	NCT02142738	Multicenter, phase 3	First-line	n.r.	PD-L1	Non-squamous and squamous	64.5/66	154/151	200 mg pembrolizumab or platinum-based chemotherapy every 3 weeks	60	Merck Sharp & Dohme

n.r., not reported; NCT, National Clinical Trial; Exp, experiment group; Con, control group; ICIs, immune checkpoint inhibitors; PD-1, programmed cell death 1; PD-L1, programmed cell death ligand 1; q3w, every 3 weeks; q2w, every 2 weeks.

### Methodological Quality of Studies

The methodological quality of trials included was high overall. Random sequence generation was adequate in all trials. Allocation concealment was not reported in all trials. Due to the open-label design of all included studies, neither investigators nor patients were masked to assign study treatments. Nevertheless, for the endpoint of OS and TRAEs, they are not likely to be susceptible to lack of blinding. Besides, we usually assume that blinding of outcome assessment was of generally low risk of bias for objective outcomes. All eligible studies demonstrated a clear patient flow or applied an intention-to-treat analysis. Therefore, there was no hint of attrition bias. Furthermore, all studies except for one provided a trial protocol, and the remaining one (22) did not report results selectively. We presented the cumulative percentages for each risk of bias domain in **Figure S1**.

### Pairwise Meta-Analysis

As for OS, all 13 trials reported information on HR values and were included for pairwise meta-analysis. HRs were explicitly reported in 12 studies (13, 18–28) and extracted from Kaplan–Meier curves in the remaining one (14). Head-to-head comparisons revealed that compared with CT, OS was improved in patients treated with atezolizumab (HR, 0.75; 95% CI, 0.69–0.83), cemiplimab (HR, 0.68; 95% CI, 0. 57–0.81), nivolumab (HR, 0.76; 95% CI, 0.62–0.93), and pembrolizumab (HR, 0.71; 95% CI, 0.62–0.82). No significant difference in OS was observed when comparing avelumab with CT (HR, 0.9; 95% CI, 0.78–1.03). The forest plot of OS for pairwise comparison results was presented in **Figure S2**.

In the analysis of TRAEs, direct comparisons supported those patients who received avelumab (OR, 0.30; 95% CI, 0.21–0.43), atezolizumab (OR, 0.31; 95% CI, 0.22–0.44), cemiplimab (OR, 0.17; 95% CI, 0.12–0.26), nivolumab (OR, 0.27; 95% CI, 0.21–0.35), and pembrolizumab (OR, 0.31; 95% CI, 0.17–0.57) had fewer TRAEs compared with CT. The forest plot of TRAEs for pairwise comparison results was presented in **Figure S3**.

### Network Meta-Analysis

As for OS, indirect comparison results were illustrated in **Figure S4A**, and atezolizumab (HR, 0.77; 95% CrI, 0.62–0.96), nivolumab (HR, 0.75; 95% CrI, 0.62–0.93), and pembrolizumab (HR, 0.71; 95% CrI, 0.57–0.89) had significantly lower HRs of OS compared with CT.

In terms of TRAEs, the incidence of TRAEs was lower with avelumab (OR, 0.30; 95% CrI, 0.11–0.77), atezolizumab (OR, 0.32; 95% CrI, 0.18–0.63), cemiplimab (OR, 0.17; 95% CrI, 0.06–0.45), nivolumab (OR, 0.27; 95% CrI, 0.16–0.44), and pembrolizumab (OR, 0.30; 95% CrI, 0.17–0.54) than that with CT. The results providing indirect comparisons between treatments are presented in **Figure S4B**.

**Figure 3:**
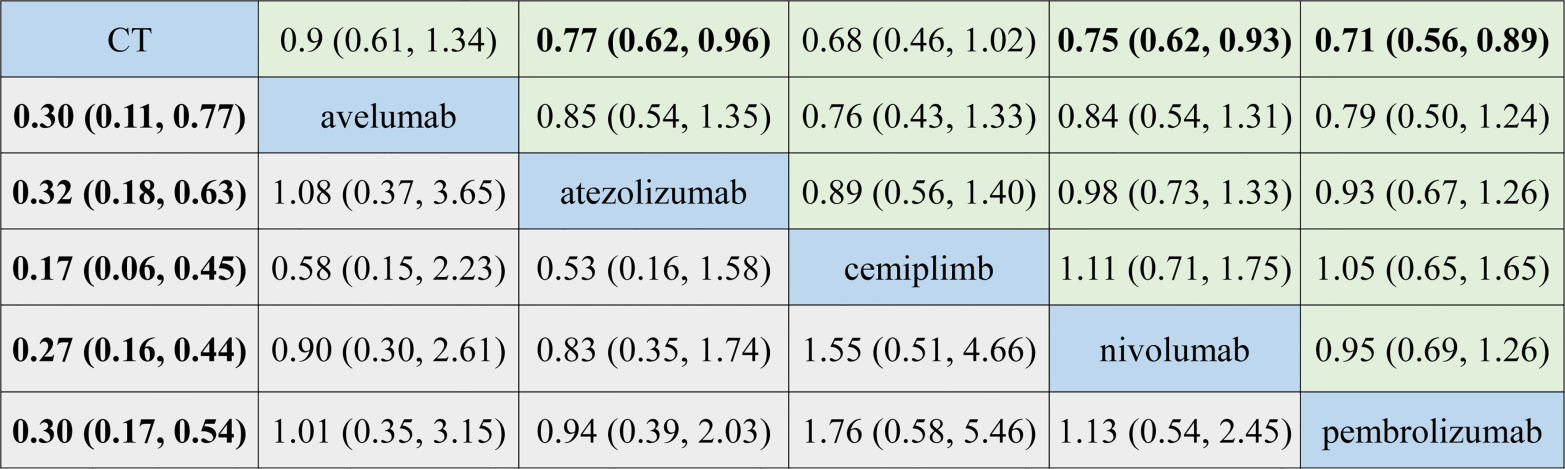
Summary for target outcomes including overall survival (OS) and treatment-related adverse events (TRAEs). The upper right section presented results for OS, and the left bottom section showed the results for TRAEs. The number in each cell represented the result that the treatment in the upper left cell divides that treatment in the lower right cell. Bold number indicates statistical significance. CT, chemotherapy.

**Figure 3** reported all pooled results of the NMA, in which the upper right section indicates the results of the OS, and the left bottom section indicates the results of TRAEs.

### Ranking of All Treatments

**Figure 4:**
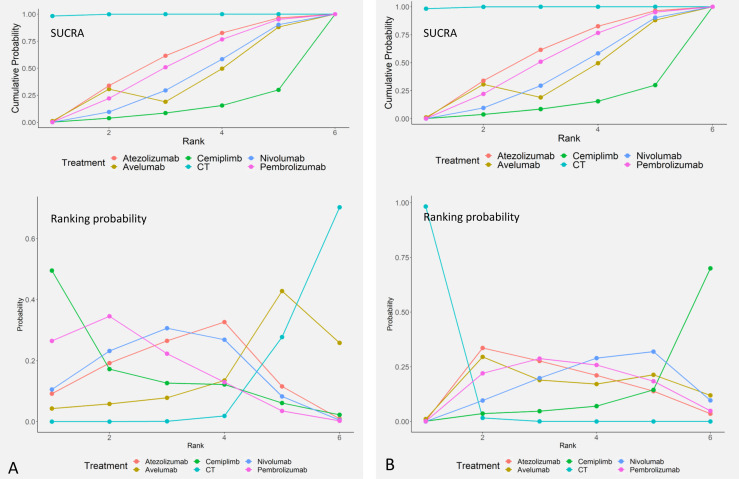
Ranking of overall survival (OS) **(A)** and treatment-related adverse events (TRAEs) **(B)**.

Comparative efficacy of treatments for OS and TRAEs based on treatment ranking probabilities was summarized in **Figures 4A, B**, respectively. Cemiplimab provided the most favorable balance between efficacy and safety. For OS, cemiplimab ranked the first (probability = 49.5%), pembrolizumab ranked the second (probability = 34.6%), and nivolumab ranked the third (probability = 30.6%). For TRAEs, CT had the highest SUCRA ranking (98.3%), and it means that CT caused the most TRAEs. Atezolizumab ranked second (52.5%), and pembrolizumab ranked third (28.8%).

## Discussion

This is the first NMA on this topic. We draw some important conclusions from this study. First, for OS, direct evidence suggested that all anti-PD-1/PD-L1 treatments except for avelumab yielded a better OS compared with CT; however, network evidence did not support cemiplimab to be superior to CT in OS. Second, for TRAEs, direct evidence indicated that patients receiving ICI monotherapies (vs. CT) were less likely to increase the risk of TRAEs. Third, no ICI has been shown to be superior to another in terms of OS and TRAEs, which were simultaneously supported by direct and network evidence. Fourth, the ranking of all target drugs was cemiplimab, pembrolizumab, nivolumab, atezolizumab, avelumab, and CT in enhancing OS. Fifth, the ranking of all regimens was CT, atezolizumab, pembrolizumab, nivolumab, avelumab, and cemiplimab in terms of TRAEs.

Cemiplimab is a high-affinity human monoclonal antibody that blocks PD-1 directly (44). Previous primary studies demonstrated that cemiplimab showed substantial antitumor activity, durable response, and an acceptable safety profile in patients with advanced cutaneous squamous cell carcinoma (45, 46). In addition, the EMPOWER-Lung 1 trial proved for the first time that cemiplimab appears to be an attractive choice for the treatment of advanced NSCLC as a first-line option. The results revealed that this ICI markedly improved OS and progression-free survival (PFS) compared with platinum-based chemotherapy (14). A cost-effectiveness analysis based on the data from the EMPOWER-Lung 1 trial found that this regimen was a cost-effective strategy at a willingness-to-pay threshold of $150,000 per quality-adjusted life-years (47). These favorable data provide support that cemiplimab represents a new option for the treatment of NSCLC.

In our study, cemiplimab should be the first choice for checkpoint inhibitor-based therapy in the general population of patients with NSCLC in the light of OS and risk of TRAEs. Two main reasons that may explain why network evidence did not find a statistically significant difference between cemiplimab and CT are the following: (a) only one clinical trial investigating the effect and safety of cemiplimab in patients with advanced NSCLC with PD-L1 of at least 50% was powered to detect only very large differences, and (b) this study had insufficient follow-up (median follow-up for cemiplimab and CT is 10.8 and 10.9 months, respectively). And it is known that immunotherapy needed a longer follow-up to determine its efficacy and safety, especially for the survival impact.

PD-L1 expression has been viewed as an effective tumor biomarker of response to PD-(L)1 inhibitor. Previous studies in NSCLC have demonstrated that higher expression of PD-L1 on tumor and/or immune cells correlated with better efficacy of anti-PD-1/PD-L1 treatments (13, 14, 21, 25, 48, 49). Thus, the extent of PD-L1 expression appears to be a somewhat continuous measure describing potential responsiveness to the PD-1 pathway blockade. One issue deserved attention for the patients with lower-level, negative, or unknown PD-L1 status: whether they should be excluding the possible benefit from ICIs. A subgroup analysis in a meta-analysis showed that patients with PD-L1 expression of <1% also derived benefit from ICIs (50). Furthermore, PD-L1 status has low sensibility (72%) and specificity (58%), thus PD-L1 status alone is not an appropriate biomarker to optimize immunotherapy. Thus, beyond PD-L1 expression, how to choose among available anti-PD-1/PD-L1 treatments in the same setting? Tumor mutation burden might serve as a promising predictive marker. Studies have shown that high mutational burden was an independent biomarker across a range of solid tumors (51, 52). Furthermore, the results of CheckMate 227 trial also showed higher tumor mutation burden has a positive correlation with response to immunotherapy in NSCLC (53).

It was worth mentioning that many original studies have investigated the clinical application of anti-PD-1/PD-L1 treatments in NSCLC. But there are no trials simultaneously investigating the efficacy and safety of all five therapeutic options (avelumab, atezolizumab, cemiplimab, nivolumab, and pembrolizumab). To date, two traditional meta-analyses were conducted to explore the efficacy of anti-PD-1/PD-L1 treatments (includes nivolumab, pembrolizumab, and atezolizumab) vs. chemotherapy in patients with NSCLCs (54, 55). And one NMA only assessed the difference in both efficacy and safety profiles among nivolumab, pembrolizumab, and atezolizumab in pretreated NSCLC patients (56). Consequently, which ICI is superior remained unclear.

Results of Zeng et al. (54) indicated that the OS rate was prolonged by anti-PD-1/PD-L1, as well as PFS. Meanwhile, the authors also demonstrated that anti-PD-1/PD-L1 could greatly enhance the objective response rate (ORR) with fewer adverse events. Shi et al. (55) supposed that patients obtained greater OS and PFS from treatments with PD-1/PD-L1 inhibitors in all levels of PD-L1 expression subgroups. The study also demonstrated that PD-1/PD-L1 inhibitor groups had a significantly lower risk in any TRAE than CT. A subgroup analysis showed that patients with PD-L1-positive advanced NSCLC had a significantly higher risk in immune-related AEs treated with PD-1/PD-L1 inhibitors compared with chemotherapy. Passiglia et al. (56) verified that nivolumab and pembrolizumab are associated with a significant increase of ORR as compared to atezolizumab, and that nivolumab is related to a significantly lower incidence of G3–5 AEs as compared to the other drugs. But they argue that no significant differences in both pneumonitis and discontinuation rate have been observed among these agents.

In contrast to previous meta-analyses and NMA, we comprehensively assessed all anti-PD-1/PD-L1 treatments that are used for the treatments of NSCLCs and thus acquired more informative findings. Firstly, direct and network evidence supported that atezolizumab, nivolumab, and pembrolizumab were associated with a better OS and obtained less TRAEs compared with CT, which were in line with previous results. Secondly, our analysis confirmed that avelumab and cemiplimab also lowered the risk of TRAEs. We deemed cemiplimb as the preferred option for patients with NSCLC combining OS and risk of TRAEs. Thirdly, it was the first time to make hierarchies of six different treatment strategies, all of which were not reported in previous studies.

We must acknowledge some limitations in this NMA. Firstly, HRs and corresponding 95% CIs were primarily extracted from the original studies, which may lead to a reporting bias. However, such kind of risk was difficult to resolve unless assessing the individual patient data. Secondly, majority of trials are short of long follow-up. This factor may affect an adequate observation for survival outcomes, especially for immunotherapy. To reduce this impact caused by follow-up, we selected those reporting the most updated data if multiple publications with different follow-ups from the same trial exist. Thirdly, two trials specified histology types (squamous or non-squamous) for registration (27, 28). Incorporating specific pathological subtypes into one population for analysis did not fully represent these trials that recruited all pathological types. Fourthly, in our study, comparisons among all ICIs are indirect, and the level of evidence was relatively low. Hence, direct evidence is warranted to verify our findings. Fifthly, there were fewer RCTs (13) of treatment for NSCLC with current findings, so further subgroup analysis and sensitivity analysis were impossible. Sixthly, the studies selected for our NMA are performed in different lines of treatment (some in first and some in second/third) and on population with different PD-L1 statuses (>50%, 1%, and unselected). However, we could not investigate the impact of these factors on the pooled results due to limited data, although line of treatment and PD-L1 status have a well-known impact on clinical outcomes of NSCLC patents.

## Conclusions

In summary, the present NMA finds that cemiplimab may have the most favorable risk–benefit ratio for NSCLC patients compared with four other target therapeutic managements. However, future research, particularly large-scale high-quality RCTs with a more mature follow-up, is required to further determine which PD-1/PD-L1 agents should be preferentially selected for this specific patient due to the limitations of this network and variations of included studies in the line of treatment and PD-L1 status.

## Data Availability Statement

The original contributions presented in the study are included in the article/**Supplementary Material**. Further inquiries can be directed to the corresponding author.

## Author Contributions

MJ contributed to conception or design. CL and DD contributed to acquisition, analysis, or interpretation of data. HT drafted the article for important content. CY critically revised the article for important intellectual content. All authors gave final approval.

## Conflict of Interest

The authors declare that the research was conducted in the absence of any commercial or financial relationships that could be construed as a potential conflict of interest.

## Publisher’s Note

All claims expressed in this article are solely those of the authors and do not necessarily represent those of their affiliated organizations, or those of the publisher, the editors and the reviewers. Any product that may be evaluated in this article, or claim that may be made by its manufacturer, is not guaranteed or endorsed by the publisher.

## Supplementary Material

Supplementary Figure 1 Risk of bias summary.

Supplementary Figure 2 Meta-analysis of OS.

Supplementary Figure 3 Meta-analysis of TRAEs.

Supplementary Figure 4 Indirect meta-analysis of OS and TRAEs when target PD-1/PD-L1 agents compared with CT.
