# Supplementary figures and images for: Comparative Efficacy and Safety of Anti-PD-1/PD-L1 for the Treatment of Non-Small Cell Lung Cancer: A Network Meta-Analysis of 13 Randomized Controlled Studies
Source: Front Oncol. 2022 May 10;12:827050. doi: 10.3389/fonc.2022.827050 (PMC9127412; doi:10.3389/fonc.2022.827050)

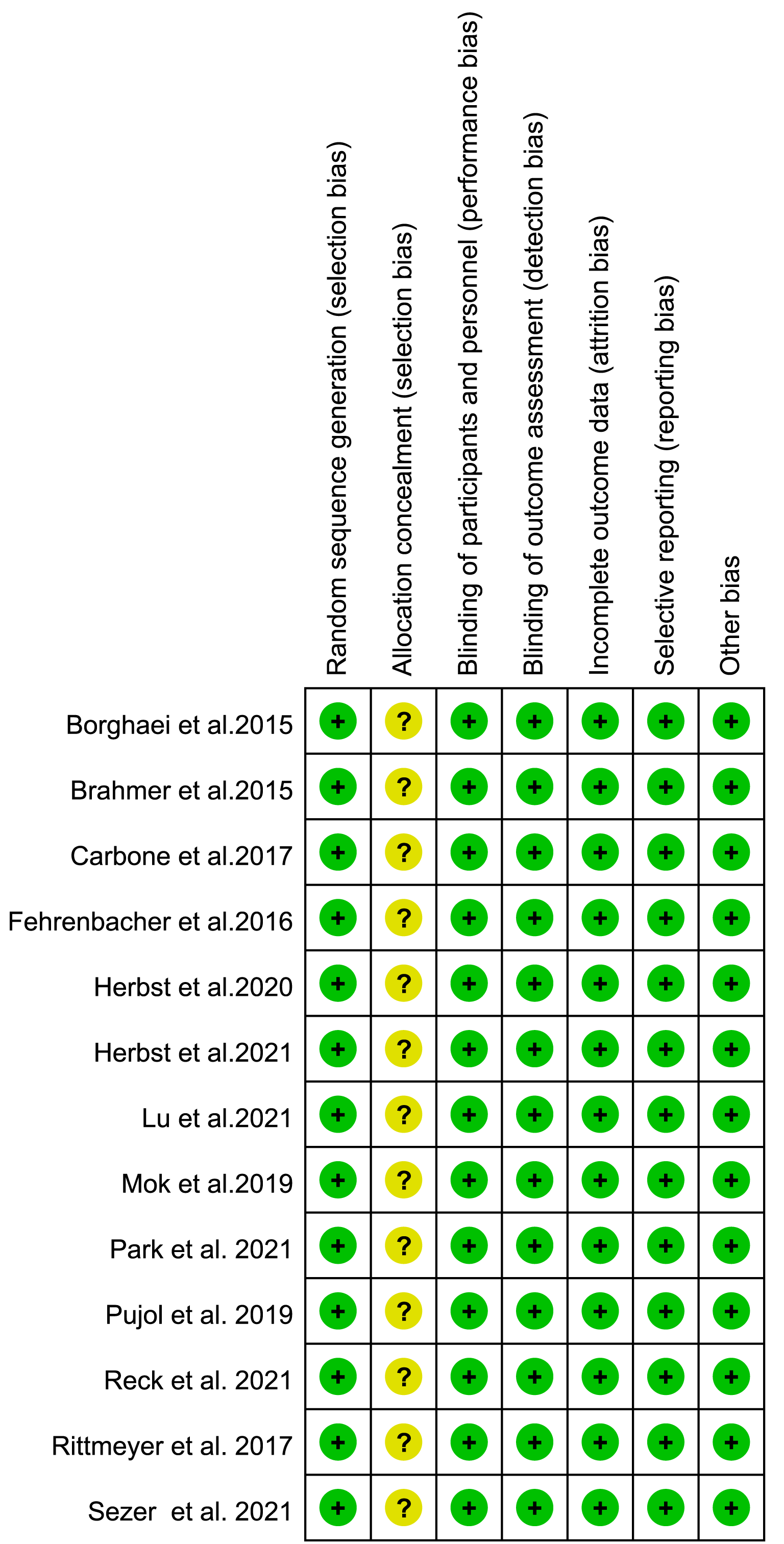

Supplement: Supplementary Figure 1 — Risk of bias summary. [file Image_1.tif]

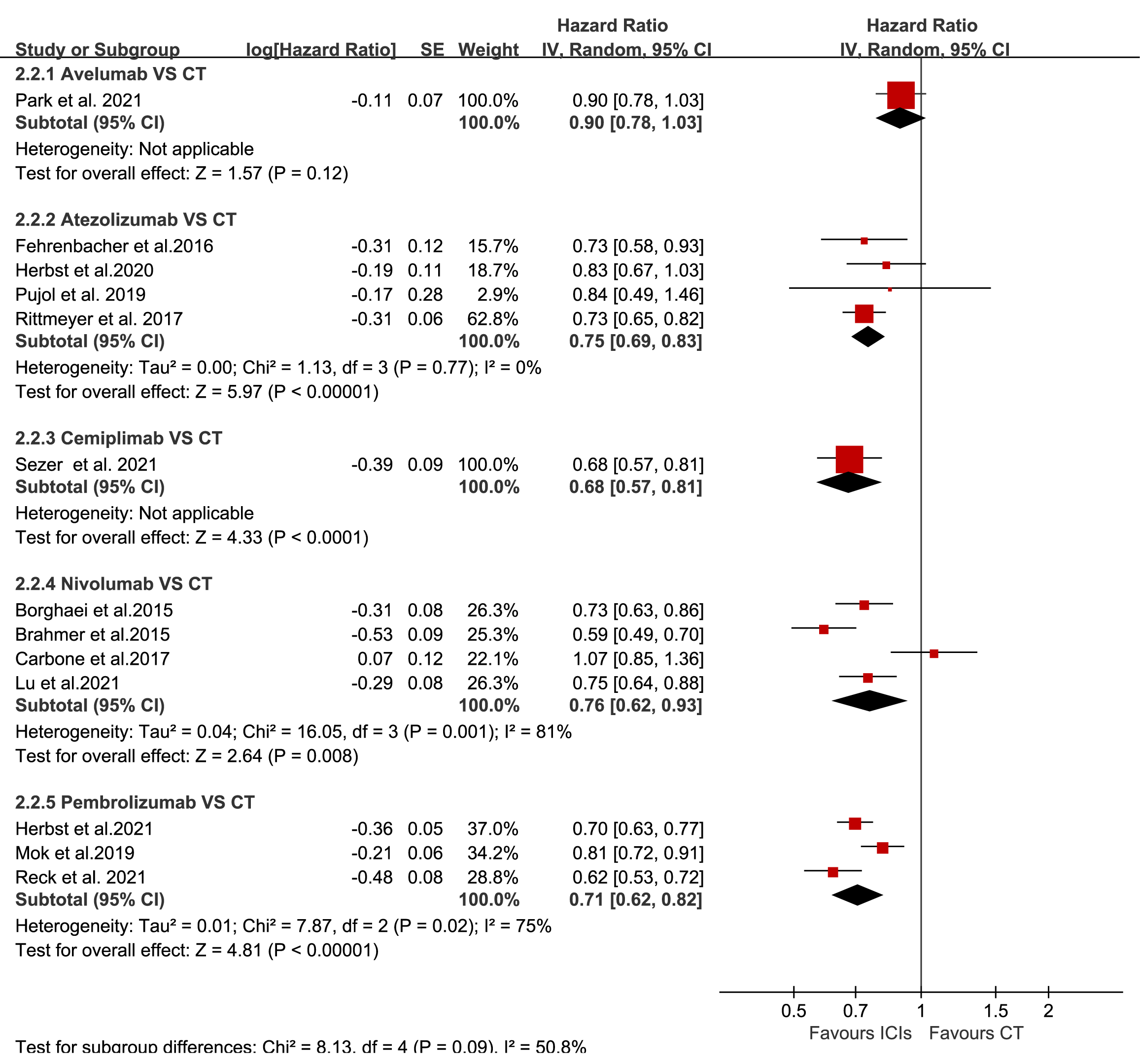

Supplement: Supplementary Figure 2 — Meta-analysis of OS. [file Image_2.tif]

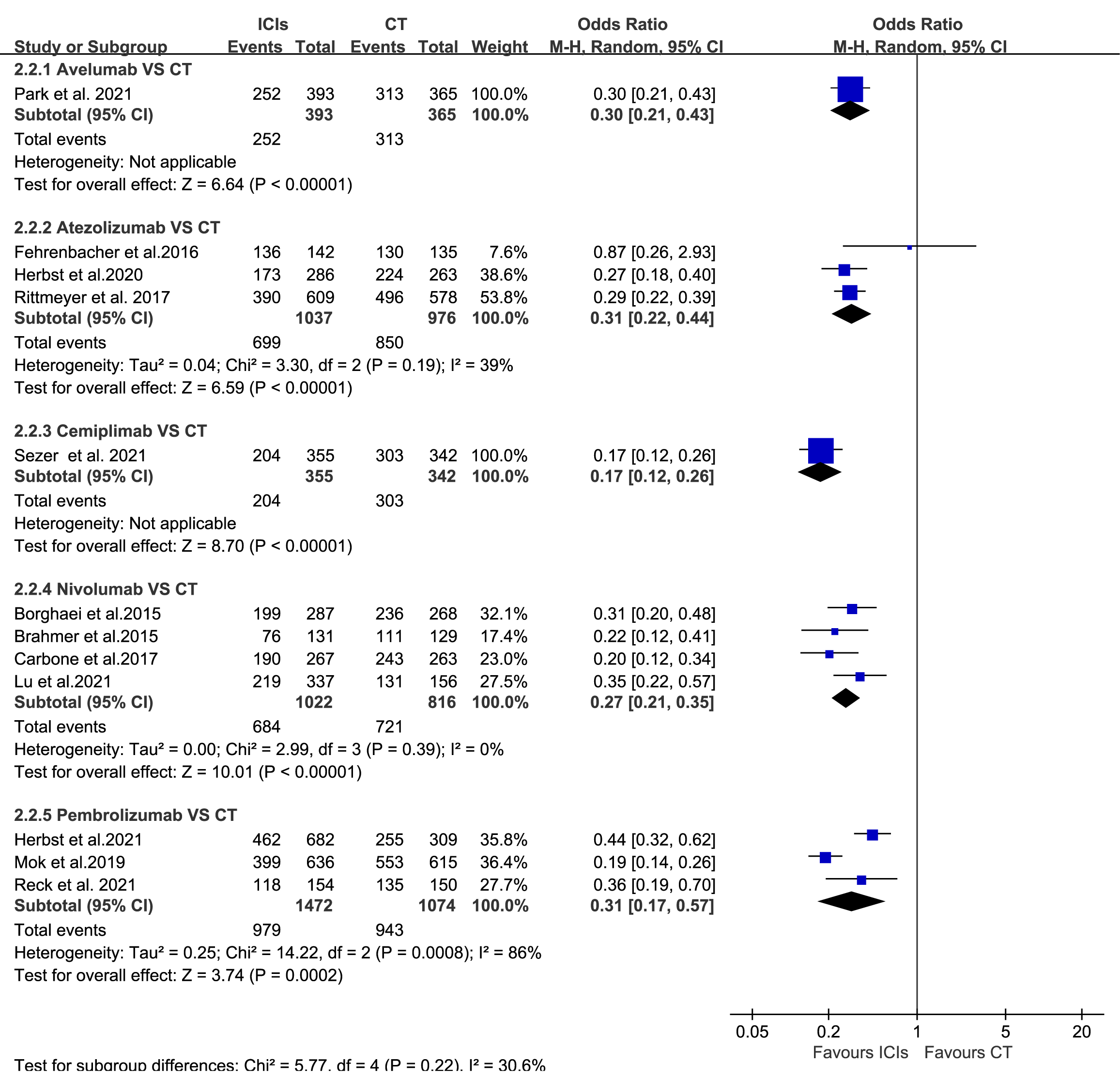

Supplement: Supplementary Figure 3 — Meta-analysis of TRAEs. [file Image_3.tif]

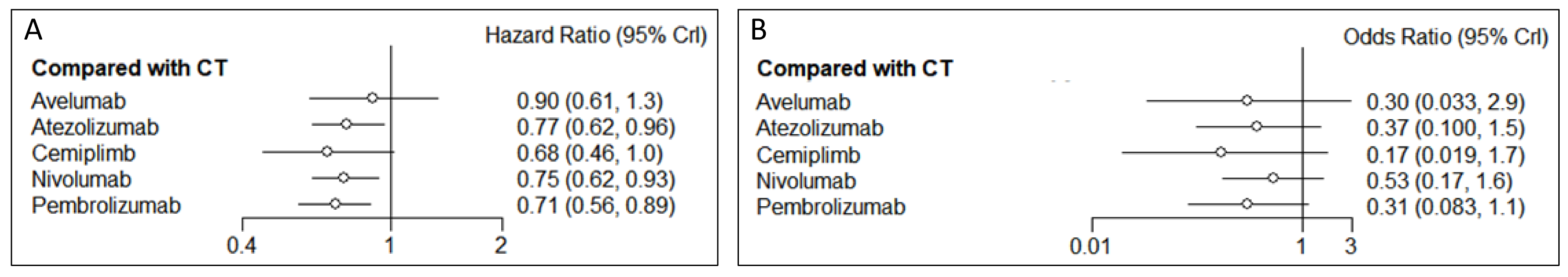

Supplement: Supplementary Figure 4 — Indirect meta-analysis of OS and TRAEs when target PD-1/PD-L1 agents compared with CT. [file Image_4.tif]
