# Supplementary material for: Comparative Efficacy and Safety of Anti-PD-1/PD-L1 for the Treatment of Non-Small Cell Lung Cancer: A Network Meta-Analysis of 13 Randomized Controlled Studies
Source: Front Oncol. 2022 May 10;12:827050. doi: 10.3389/fonc.2022.827050 (PMC9127412; doi:10.3389/fonc.2022.827050)
Supplement: Supplementary file 6 [file Table_2.docx]

| **Table S2. Search strategy of PubMed** | |
| --- | --- |
| NO. | Query |
| 31 | #1 AND #28 AND #29 AND #30 |
| 30 | "Randomized Controlled Trial"[Publication Type] OR "Randomized Controlled Trials as Topic"[MeSH Terms] OR "Controlled Clinical Trial"[Publication Type] OR "Controlled Clinical Trials as Topic"[MeSH Terms] OR "clinical trials, phase iii as topic"[MeSH Terms] OR "clinical trials, phase iv as topic"[MeSH Terms] OR "random*"[Title/Abstract] OR "clinical trials phase iii"[Title/Abstract] OR "clinical trials phase iv"[Title/Abstract] |
| 29 | "drug related side effects and adverse reactions"[MeSH Terms] OR "adverse drug reaction*"[Title/Abstract] OR "adverse drug event*"[Title/Abstract] OR "drug toxicit*"[Title/Abstract] OR "adverse event*"[Title/Abstract] OR "side effect*"[Title/Abstract] OR "Safety"[Title/Abstract] OR "Security"[Title/Abstract] OR "Complications"[Title/Abstract] OR "Harms"[Title/Abstract] |
| 28 | #2 OR #3 OR #4 OR #5 OR #6 OR #7OR #8 OR #9 OR #10 OR #11 OR #12 OR #13 OR #14 OR #15 OR #16 OR #17 OR #18 OR #19 OR #20 OR #21 OR #22 OR #23 OR #24 OR #25 OR #26 OR #27 |
| 27 | "ticilimumab"[Title/Abstract] OR "CP-675"[Title/Abstract] OR ("CP675"[All Fields] AND "cpd"[Title/Abstract]) OR "CP-675"[Title/Abstract] OR "CP-675206"[Title/Abstract] OR "CP675206"[Title/Abstract] OR "CP-675206"[Title/Abstract] |
| 26 | "tremelimumab"[Supplementary Concept] |
| 25 | "SCH-900475"[Title/Abstract] OR "Keytruda"[Title/Abstract] OR "MK-3475"[Title/Abstract] OR "lambrolizumab"[Title/Abstract] |
| 24 | "pembrolizumab"[Supplementary Concept] |
| 23 | "CT-011"[Title/Abstract]" |
| 22 | "pidilizumab"[Supplementary Concept] |
| 21 | "Opdivo"[Title/Abstract] OR "ONO-4538"[Title/Abstract] OR "ONO-4538"[Title/Abstract] OR "ONO4538"[Title/Abstract] OR "MDX-1106"[Title/Abstract] OR "MDX-1106"[Title/Abstract] OR "MDX1106"[Title/Abstract] OR "BMS-936558"[Title/Abstract] OR "BMS-936558"[Title/Abstract] OR "BMS936558"[Title/Abstract] |
| 20 | "Nivolumab"[MeSH Terms] |
| 19 | "Yervoy"[Title/Abstract] OR "MDX-010"[Title/Abstract] OR "MDX010"[Title/Abstract] OR "MDX-010"[Title/Abstract] OR "MDX-CTLA-4"[Title/Abstract] OR "MDX-CTLA-4"[Title/Abstract] |
| 18 | "Ipilimumab"[MeSH Terms] |
| 17 | "MEDI4736"[Title/Abstract] OR "MEDI-4736"[Title/Abstract] OR "Imfinzi"[Title/Abstract] |
| 16 | "durvalumab"[Supplementary Concept] |
| 15 | "REGN2810"[Title/Abstract] |
| 14 | "cemiplimab"[Supplementary Concept] |
| 13 | "MSB0010682"[Title/Abstract] OR "bavencio"[Title/Abstract] OR "MSB0010718C"[Title/Abstract] OR "MSB-0010718C"[Title/Abstract] |
| 12 | "avelumab"[Supplementary Concept] |
| 11 | "anti-PDL1"[Title/Abstract] OR "MPDL3280A"[Title/Abstract] OR "MPDL-3280A"[Title/Abstract] OR "Tecentriq"[Title/Abstract] OR "RG7446"[Title/Abstract] OR "RG-7446"[Title/Abstract] |
| 10 | "atezolizumab"[Supplementary Concept] |
| 9 | "cytotoxic t lymphocyte associated antigen 4"[Title/Abstract] OR "cytotoxic t lymphocyte associated antigen 4"[Title/Abstract] OR "cd152 antigen*"[Title/Abstract] OR "ctla 4 protein"[Title/Abstract] OR "cytotoxic t lymphocyte antigen 4"[Title/Abstract] OR "cytotoxic t lymphocyte antigen 4"[Title/Abstract] |
| 8 | "CTLA-4 Antigen"[MeSH Terms] |
| 7 | "b7 h1 antigen"[Title/Abstract] OR "b7 h1 antigen"[Title/Abstract] OR "programmed death ligand 1"[Title/Abstract] OR "cd274 antigen*"[Title/Abstract] OR (("B7-H1"[All Fields] AND ("immune"[All Fields] OR "immuned"[All Fields] OR "immunes"[All Fields] OR "immunisation"[All Fields] OR "vaccination"[MeSH Terms] OR "vaccination"[All Fields] OR "immunization"[All Fields] OR "immunization"[MeSH Terms] OR "immunisations"[All Fields] OR "immunizations"[All Fields] OR "immunise"[All Fields] OR "immunised"[All Fields] OR "immuniser"[All Fields] OR "immunisers"[All Fields] OR "immunising"[All Fields] OR "immunities"[All Fields] OR "immunity"[MeSH Terms] OR "immunity"[All Fields] OR "immunization s"[All Fields] OR "immunize"[All Fields] OR "immunized"[All Fields] OR "immunizer"[All Fields] OR "immunizers"[All Fields] OR "immunizes"[All Fields] OR "immunizing"[All Fields])) AND "costimulatory protein"[Title/Abstract]) OR (("B7"[All Fields] AND "H1"[All Fields] AND ("immune"[All Fields] OR "immuned"[All Fields] OR "immunes"[All Fields] OR "immunisation"[All Fields] OR "vaccination"[MeSH Terms] OR "vaccination"[All Fields] OR "immunization"[All Fields] OR "immunization"[MeSH Terms] OR "immunisations"[All Fields] OR "immunizations"[All Fields] OR "immunise"[All Fields] OR "immunised"[All Fields] OR "immuniser"[All Fields] OR "immunisers"[All Fields] OR "immunising"[All Fields] OR "immunities"[All Fields] OR "immunity"[MeSH Terms] OR "immunity"[All Fields] OR "immunization s"[All Fields] OR "immunize"[All Fields] OR "immunized"[All Fields] OR "immunizer"[All Fields] OR "immunizers"[All Fields] OR "immunizes"[All Fields] OR "immunizing"[All Fields])) AND "costimulatory protein"[Title/Abstract]) OR (("B7H1"[All Fields] AND ("immune"[All Fields] OR "immuned"[All Fields] OR "immunes"[All Fields] OR "immunisation"[All Fields] OR "vaccination"[MeSH Terms] OR "vaccination"[All Fields] OR "immunization"[All Fields] OR "immunization"[MeSH Terms] OR "immunisations"[All Fields] OR "immunizations"[All Fields] OR "immunise"[All Fields] OR "immunised"[All Fields] OR "immuniser"[All Fields] OR "immunisers"[All Fields] OR "immunising"[All Fields] OR "immunities"[All Fields] OR "immunity"[MeSH Terms] OR "immunity"[All Fields] OR "immunization s"[All Fields] OR "immunize"[All Fields] OR "immunized"[All Fields] OR "immunizer"[All Fields] OR "immunizers"[All Fields] OR "immunizes"[All Fields] OR "immunizing"[All Fields])) AND "costimulatory protein"[Title/Abstract]) OR "pd l1 costimulatory protein"[Title/Abstract] OR "pd l1 costimulatory protein"[Title/Abstract] OR "programmed cell death 1 ligand 1 protein"[Title/Abstract] OR "pd l1 protein"[Title/Abstract] OR "pd l1 protein"[Title/Abstract] OR "programmed cell death 1 ligand 1"[Title/Abstract] |
| 6 | "B7-H1 Antigen"[MeSH Terms] |
| 5 | "programmed cell death 1 receptor"[Title/Abstract] OR "pd 1 protein"[Title/Abstract] OR "pd 1 protein"[Title/Abstract] OR "pd 1 receptor"[Title/Abstract] OR "pd 1 receptor"[Title/Abstract] OR ("CD279"[All Fields] AND "antigen*"[Title/Abstract]) OR "pd1 receptor"[Title/Abstract] OR "programmed cell death protein 1"[Title/Abstract] OR "programmed cell death 1 protein"[Title/Abstract] |
| 4 | "Programmed Cell Death 1 Receptor"[MeSH Terms] |
| 3 | "immune checkpoint inhibitor*"[Title/Abstract] OR "immune checkpoint blockers"[Title/Abstract] OR "immune checkpoint blockade"[Title/Abstract] OR "immune checkpoint inhibition"[Title/Abstract] OR "pd l1 inhibitor*"[Title/Abstract] OR "pd l1 inhibitor*"[Title/Abstract] OR "programmed death ligand 1 inhibitors"[Title/Abstract] OR "programmed death ligand 1 inhibitors"[Title/Abstract] OR "ctla 4 inhibitor*"[Title/Abstract] OR "cytotoxic t lymphocyte associated protein 4 inhibitor*"[Title/Abstract] OR "cytotoxic t lymphocyte associated protein 4 inhibitor*"[Title/Abstract] OR "pd 1 inhibitor*"[Title/Abstract] OR "pd 1 inhibitor*"[Title/Abstract] OR "programmed cell death protein 1 inhibitor*"[Title/Abstract] OR "pd 1 pd l1 blockade"[Title/Abstract] OR "pd 1 pd l1 blockade"[Title/Abstract] |
| 2 | "Immune Checkpoint Inhibitors"[MeSH Terms] |
| 1 | "carcinoma, non small cell lung"[MeSH Terms] OR "non small cell lung carcinoma*"[Title/Abstract] OR "non small cell lung carcinoma"[Title/Abstract] OR "non small cell lung carcinoma"[Title/Abstract] OR "non small cell lung cancer"[Title/Abstract] OR "nonsmall cell lung cancer"[Title/Abstract] |

**Search strategy of Embase**

| No. | Query |
| --- | --- |
| #35 | #27 AND #30 AND #33 AND #34 |
| #34 | random* |
| #33 | #31 OR #32 |
| #32 | 'adverse drug effect':ti,ab,kw OR 'adverse drug event':ti,ab,kw OR 'drug adverse effect':ti,ab,kw OR 'drug adverse reaction':ti,ab,kw OR 'drug side effect':ti,ab,kw OR ('drug -related side effects':ti,ab,kw AND 'adverse reactions':ti,ab,kw) OR 'long term adverse effects':ti,ab,kw OR ('metabolic side effects of drugs':ti,ab,kw AND substances:ti,ab,kw) |
| #31 | 'adverse drug reaction'/exp |
| #30 | #28 OR #29 |
| #29 | 'bronchial non small cell cancer':ti,ab,kw OR 'non-small-cell lung carcinoma':ti,ab,kw OR 'bronchial non small cell carcinoma':ti,ab,kw OR 'lung non small cell cancer':ti,ab,kw OR 'lung non small cell carcinoma':ti,ab,kw OR 'non small cell bronchial cancer':ti,ab,kw OR 'non small cell lung carcinoma':ti,ab,kw OR 'non small cell pulmonary cancer':ti,ab,kw OR 'non small cell pulmonary carcinoma':ti,ab,kw OR 'pulmonary non small cell cancer':ti,ab,kw OR 'pulmonary non small cell carcinoma':ti,ab,kw |
| #28 | 'non small cell lung cancer'/exp |
| #27 | #1 OR #2 OR #3 OR #4 OR #5 OR #6 OR #7 OR #8 OR #9 OR #10 OR #11 OR #12 OR #13 OR #14 OR #15 OR #16 OR #17 OR #18 OR #19 OR #20 OR #21 OR #22 OR #23 OR #24 OR #25 OR #26 |
| #26 | 'cp 675 206':ti,ab,kw OR 'cp 675206':ti,ab,kw OR 'cp675 206':ti,ab,kw OR cp675206:ti,ab,kw OR tremelimumab:ti,ab,kw |
| #25 | 'ticilimumab'/exp |
| #24 | keytruda:ti,ab,kw OR lambrolizumab:ti,ab,kw OR 'mk 3475mk3475':ti,ab,kw OR mk3475:ti,ab,kw OR 'sch 900475':ti,ab,kw OR sch900475:ti,ab,kw |
| #23 | 'pembrolizumab'/exp |
| #22 | 'ct 011':ti,ab,kw OR ct011:ti,ab,kw |
| #21 | 'pidilizumab'/exp |
| #20 | 'bms 936558':ti,ab,kw OR bms936558:ti,ab,kw OR 'cmab 819':ti,ab,kw OR cmab819:ti,ab,kw OR 'mdx 1106':ti,ab,kw OR mdx1106:ti,ab,kw OR 'ono 4538':ti,ab,kw OR ono4538:ti,ab,kw OR opdivo:ti,ab,kw |
| #19 | 'nivolumab'/exp |
| #18 | 'bms 734016':ti,ab,kw OR bms734016:ti,ab,kw OR 'mdx 010':ti,ab,kw OR 'mdx 101':ti,ab,kw OR mdx010:ti,ab,kw OR mdx101:ti,ab,kw OR strentarga:ti,ab,kw OR yervoy:ti,ab,kw |
| #17 | 'ipilimumab'/exp |
| #16 | imfinzi:ti,ab,kw OR 'medi 4736':ti,ab,kw OR medi4736:ti,ab,kw |
| #15 | 'durvalumab'/exp |
| #14 | cemiplimab:ti,ab,kw OR 'cemiplimab rwlc':ti,ab,kw OR libtayo:ti,ab,kw OR 'regn 2810':ti,ab,kw OR regn2810:ti,ab,kw OR 'sar 439684':ti,ab,kw OR sar439684:ti,ab,kw |
| #13 | 'cemiplimab'/exp |
| #12 | bavencio:ti,ab,kw OR 'msb 0010682':ti,ab,kw OR 'msb 0010718c':ti,ab,kw OR 'msb 10682':ti,ab,kw OR 'msb 10718c':ti,ab,kw OR msb0010682:ti,ab,kw OR msb0010718c:ti,ab,kw OR msb10682:ti,ab,kw OR msb10718c:ti,ab,kw OR 'pf 06834635':ti,ab,kw OR 'pf 6834635':ti,ab,kw OR pf06834635:ti,ab,kw OR pf6834635:ti,ab,kw |
| #11 | 'avelumab'/exp |
| #10 | 'rg 7446':ti,ab,kw OR rg7446:ti,ab,kw OR 'mpdl 3280a':ti,ab,kw OR mpdl3280a:ti,ab,kw OR tecentriq:ti,ab,kw OR tecntriq:ti,ab,kw |
| #9 | 'atezolizumab'/exp |
| #8 | 'antigen cd152':ti,ab,kw OR 'cd152 antigen':ti,ab,kw OR 'ctla 4':ti,ab,kw OR 'ctla-4 antigen':ti,ab,kw OR ctla4:ti,ab,kw OR 'cytotoxic t lymphocyte associated antigen 4':ti,ab,kw |
| #7 | 'cytotoxic t lymphocyte antigen 4'/exp |
| #6 | 'antigen b7 h1':ti,ab,kw OR 'antigen b7h1':ti,ab,kw OR 'antigen cd274':ti,ab,kw OR 'b7 h1 antigen':ti,ab,kw OR 'b7 h1 protein':ti,ab,kw OR 'b7 homolog 1 protein':ti,ab,kw OR 'b7-h1 antigen':ti,ab,kw OR 'b7h1 antigencd274 antigen':ti,ab,kw OR 'b7h1 protein':ti,ab,kw OR 'cd274 antigen':ti,ab,kw OR 'cd274 antigens':ti,ab,kw OR 'pdcd1 ligand 1':ti,ab,kw OR 'pdcd1lg1 protein':ti,ab,kw OR 'programmed cell death 1 ligand 1':ti,ab,kw OR 'programmed death 1 ligand 1 protein':ti,ab,kw OR 'programmed death ligand 1':ti,ab,kw OR 'protein b7 h1':ti,ab,kw OR 'protein b7h1':ti,ab,kw OR 'protein pdcd1lg1':ti,ab,kw |
| #5 | 'programmed death 1 ligand 1'/exp |
| #4 | 'antigen cd279':ti,ab,kw OR 'cd279 antigen':ti,ab,kw OR 'pd 1 protein':ti,ab,kw OR 'programmed cell death 1 protein':ti,ab,kw OR 'programmed cell death 1 receptor':ti,ab,kw OR 'programmed cell death protein 1':ti,ab,kw OR 'programmed death 1 protein':ti,ab,kw OR 'programmed death protein 1':ti,ab,kw OR 'pdcd1 protein':ti,ab,kw OR 'protein pd 1':ti,ab,kw OR 'protein pdcd1':ti,ab,kw OR 'protein programmed cell death 1':ti,ab,kw OR 'protein programmed death 1':ti,ab,kw |
| #3 | 'programmed death 1 receptor'/exp |
| #2 | 'immune checkpoint blocker; immune checkpoint inhibitor*':ti,ab,kw OR 'immune checkpoint inhibitor*':ti,ab,kw |
| #1 | 'immune checkpoint inhibitor'/exp |

**Search strategy of the Cochrane library**

| ID | Search |
| --- | --- |
| #1 | MeSH descriptor: [Immune Checkpoint Inhibitors] explode all trees |
| #2 | (Programmed Cell Death Protein 1 Inhibitor*):ti,ab,kw OR (PD-1 Inhibitor*):ti,ab,kw OR (PD 1 Inhibitor*):ti,ab,kw OR (PD L1 Inhibitor*):ti,ab,kw OR (PD-L1 Inhibitor*):ti,ab,kw OR (Immune Checkpoint Blockers):ti,ab,kw OR (Immune Checkpoint Inhibitor):ti,ab,kw OR (Immune Checkpoint Blockade):ti,ab,kw OR (Immune Checkpoint Inhibition):ti,ab,kw OR (Programmed Death-Ligand 1 Inhibitors):ti,ab,kw OR (Programmed Death Ligand 1 Inhibitors):ti,ab,kw OR (PD 1 PD L1 Blockade):ti,ab,kw OR (Cytotoxic T-Lymphocyte-Associated Protein 4 Inhibitor*):ti,ab,kw OR (Cytotoxic T Lymphocyte Associated Protein 4 Inhibitor*):ti,ab,kw OR (CTLA-4 Inhibitor*):ti,ab,kw OR (CTLA 4 Inhibitor*):ti,ab,kw (Word variations have been searched) |
| #3 | MeSH descriptor: [Programmed Cell Death 1 Receptor] explode all trees |
| #4 | (Programmed Cell Death 1 Protein):ti,ab,kw OR (PD-1 Protein):ti,ab,kw OR (PD-1 Receptor):ti,ab,kw OR (PD1 Receptor):ti,ab,kw OR (PD 1 Receptor):ti,ab,kw OR (CD279 Antigen*):ti,ab,kw OR (PD 1 Protein):ti,ab,kw OR (Programmed Cell Death Protein 1  ):ti,ab,kw (Word variations have been searched) |
| #5 | MeSH descriptor: [B7-H1 Antigen] explode all trees |
| #6 | (Programmed Death Ligand 1):ti,ab,kw OR (Programmed Cell Death 1 Ligand 1):ti,ab,kw OR (Programmed Cell Death 1 Ligand 1 Protein):ti,ab,kw OR (PD-L1 Protein):ti,ab,kw OR (PD L1 Protein):ti,ab,kw OR (PD-L1 Costimulatory Protein):ti,ab,kw OR (PD L1 Costimulatory Protein):ti,ab,kw OR (B7 H1 Antigen):ti,ab,kw OR (B7 H1 Immune Costimulatory Protein):ti,ab,kw OR (B7H1 Immune Costimulatory Protein):ti,ab,kw OR (CD274 Antigen*):ti,ab,kw (Word variations have been searched) |
| #7 | MeSH descriptor: [CTLA-4 Antigen] explode all trees |
| #8 | (CTLA-4 Protein):ti,ab,kw OR (CTLA 4 Antigen):ti,ab,kw OR (CTLA 4 Protein):ti,ab,kw OR (Cytotoxic T-Lymphocyte Antigen 4):ti,ab,kw OR (Cytotoxic T Lymphocyte Antigen 4):ti,ab,kw OR (Cytotoxic T-Lymphocyte-Associated Antigen 4):ti,ab,kw OR (Cytotoxic T Lymphocyte Associated Antigen 4):ti,ab,kw OR (CD152 Antigen*):ti,ab,kw (Word variations have been searched) |
| #9 | (atezolizumab):ti,ab,kw OR (anti-PDL1):ti,ab,kw OR (MPDL3280A):ti,ab,kw OR (MPDL-3280A):ti,ab,kw OR (Tecentriq):ti,ab,kw OR (RG7446):ti,ab,kw OR (RG-7446):ti,ab,kw (Word variations have been searched) |
| #10 | (avelumab):ti,ab,kw OR (MSB0010682):ti,ab,kw OR (bavencio):ti,ab,kw OR (MSB0010718C):ti,ab,kw OR (MSB-0010718C):ti,ab,kw OR (cemiplimab):ti,ab,kw OR (REGN2810):ti,ab,kw OR (durvalumab):ti,ab,kw OR (MEDI4736):ti,ab,kw OR (MEDI-4736):ti,ab,kw OR (Imfinzi):ti,ab,kw (Word variations have been searched) |
| #11 | MeSH descriptor: [ipilimumab] explode all trees |
| #12 | (Ipilimumab):ti,ab,kw OR (MDX-CTLA-4):ti,ab,kw OR ( MDX CTLA 4):ti,ab,kw OR (Yervoy):ti,ab,kw OR (MDX 010):ti,ab,kw OR (MDX-010):ti,ab,kw OR (MDX010):ti,ab,kw (Word variations have been searched) |
| #13 | MeSH descriptor: [Nivolumab] explode all trees |
| #14 | (MS-936558):ti,ab,kw OR (BMS 936558):ti,ab,kw OR (BMS936558):ti,ab,kw OR ( Opdivo):ti,ab,kw OR (ONO-4538):ti,ab,kw OR (ONO4538):ti,ab,kw OR (ONO 4538):ti,ab,kw OR (MDX 1106):ti,ab,kw OR (MDX-1106):ti,ab,kw OR (MDX1106):ti,ab,kw (Word variations have been searched) |
| #15 | (pidilizumab):ti,ab,kw OR (CT-011):ti,ab,kw OR (CT 011):ti,ab,kw OR (pembrolizumab):ti,ab,kw OR (SCH-900475):ti,ab,kw OR (Keytruda):ti,ab,kw OR (MK-3475):ti,ab,kw OR (lambrolizumab):ti,ab,kw OR (tremelimumab):ti,ab,kw OR (ticilimumab):ti,ab,kw OR (CP 675):ti,ab,kw OR (CP675 cpd):ti,ab,kw OR (CP-675):ti,ab,kw OR (CP-675206):ti,ab,kw OR (CP675206):ti,ab,kw OR (CP 675206):ti,ab,kw (Word variations have been searched) |
| #16 | #1 OR #2 OR #3 OR #4 OR #5 OR #6 OR #7 #8 OR #9 OR #10 OR #11 OR #12 OR #13 OR #14 OR #15 |
| #17 | MeSH descriptor: [Carcinoma, Non-Small-Cell Lung] explode all trees |
| #18 | (Non Small Cell Lung Carcinoma):ti,ab,kw OR (Non-Small-Cell Lung Carcinoma*):ti,ab,kw OR (Non-Small Cell Lung Cancer):ti,ab,kw OR (Non-Small Cell Lung Carcinoma):ti,ab,kw OR (Nonsmall Cell Lung Cancer):ti,ab,kw (Word variations have been searched) |
| #19 | #17 OR #18 |
| #20 | MeSH descriptor: [Drug-Related Side Effects and Adverse Reactions] explode all trees |
| #21 | (Drug Toxicit*):ti,ab,kw OR (Adverse Drug Event*):ti,ab,kw OR (Adverse Drug Reaction*):ti,ab,kw OR (Side Effect*):ti,ab,kw OR (Drug Related Side Effects and Adverse Reactions):ti,ab,kw OR (Safety):ti,ab,kw OR (Security):ti,ab,kw OR (Complications):ti,ab,kw OR (Harms):ti,ab,kw (Word variations have been searched) |
| #22 | #20 OR #21 |
| #23 | (random*):ti,ab,kw (Word variations have been searched) |
| #24 | #16 AND #19 AND #22 AND #23 |
